# Novel allelic variant of *Lpa1* gene associated with a significant reduction in seed phytic acid content in rice (*Oryza sativa* L.)

**DOI:** 10.1371/journal.pone.0209636

**Published:** 2019-03-14

**Authors:** D. S. Kishor, Choonseok Lee, Dongryung Lee, Jelli Venkatesh, Jeonghwan Seo, Joong Hyoun Chin, Zhuo Jin, Soon-Kwan Hong, Jin-Kwan Ham, Hee Jong Koh

**Affiliations:** 1 Department of Plant Science, Plant Genomics and Breeding Institute, and Research Institute of Agriculture and Life Science, Seoul National University, Seoul, Republic of Korea; 2 Graduate School of Integrated Bioindustry, Sejong University, Seoul, Republic of Korea; 3 Division of Biotechnology, Kangwon National University, Chuncheon, Gangwon-do, Republic of Korea; 4 Gangwon provincial Agricultural Research & Extension Services, Chuncheon, Gangwon-do, Republic of Korea; Kyung Hee University, REPUBLIC OF KOREA

## Abstract

In plants, *myo*-inositol-1,2,3,4,5,6-hexa*kis*phosphate (InsP_6_), also known as phytic acid (PA), is a major component of organic phosphorus (P), and accounts for up to 85% of the total P in seeds. In rice (*Oryza sativa* L.), PA mainly accumulates in rice bran, and chelates mineral cations, resulting in mineral deficiencies among brown rice consumers. Therefore, considerable efforts have been focused on the development of low PA (LPA) rice cultivars. In this study, we performed genetic and molecular analyses of *OsLpa*1, a major PA biosynthesis gene, in Sanggol, a low PA mutant variety developed via chemical mutagenesis of Ilpum rice cultivar. Genetic segregation and sequencing analyses revealed that a recessive allele, *lpa1-3*, at the *OsLpa*1 locus (Os02g0819400) was responsible for a significant reduction in seed PA content in Sanggol. The *lpa1-3* gene harboured a point mutation (C623T) in the fourth exon of the predicted coding region, resulting in threonine (Thr) to isoleucine (Ile) amino acidsubstitution at position 208 (Thr208Ile). Three-dimensional analysis of Lpa1 protein structure indicated that *myo*-inositol 3-monophosphate [Ins(3)P_1_] could bind to the active site of Lpa1, with ATP as a cofactor for catalysis. Furthermore, the presence of Thr208 in the loop adjacent to the entry site of the binding pocket suggests that Thr208Ile substitution is involved in regulating enzyme activity via phosphorylation. Therefore, we propose that Thr208Ile substitution in *lpa1-3* reduces Lpa1 enzyme activity in Sanggol, resulting in reduced PA biosynthesis.

## Introduction

In most cereal crops, *myo*-inositol-1,2,3,4,5,6-hexa*kis*phosphate (InsP_6_), also known as phytic acid (PA), is considered a major source of phosphorus (P) available in the form of phytate, and accounts for 65–85% of the total P in seeds [1]. Monogastric animals poorly digest PA, as they lack the phytase enzyme, which is responsible for the release of phosphate residues [2]. PA is an efficient chelator of mineral cations, such as zinc (Zn^2+^), iron (Fe^2+^), magnesium (Mg^2+^), potassium (K^2+^), and calcium (Ca^2+^), in the nutritional tract. Because of these attributes, PA is considered as an antinutrient [3, 4]. Hence, there is a need to develop low PA (LPA) crop cultivars to maximize the nutritional benefits of grains.

Mutants associated with the LPA phenotype have been identified in several crop plants including maize (*Zea mays*) [5, 6], barley (*Hordeum vulgare*) [7], soyabean (*Glycine max*) [8], rice (*Oryza sativa*) [9], and wheat (*Triticum aestivum*) [10]. Although, LPA mutants are identified primarily on the basis of percentage reduction of PA and high inorganic P (P_i_) content in seeds [5, 11], some mutants show a significant accumulation of *myo*-inositol and inositol phosphate [Ins(1,3,4)P_3_ 5-/6] intermediates in seeds [12, 13].

Previously, the LPA phenotype of seeds has been associated with reduced agronomic performance of mutant crop plants in the field [5, 14]. It is important to understand the genetic and molecular bases of reduced agronomic performance of LPA mutants for effective utilization in breeding programs. In addition, studies show that climate change and elevated carbon dioxide (CO_2_) levels negatively affect micronutrient bioavailability and total P in grains [15, 16]. Therefore, developing crop cultivars with increased micronutrient bioavailability in seeds and greater adaptability to environmental variations, by reducing the PA content in grains, is an important priority of breeding programs.

PA is biosynthesized via two different routes: lipid dependent and lipid independent [3, 17]. The lipid dependent pathway operates in all plant organs, whereas the lipid independent pathway is predominant only in seeds [13, 17, 18]. In the first step of PA biosynthesis, D-glucose-6-phosphate is converted to *myo*-inositol 3-monophosphate [Ins(3)P_1_] by *myo*-inositol 3-phosphate synthase (MIPS) [19]. This is followed by the sequential phosphorylation of specific inositol to InsP_6_ through the action of various inositol phosphate kinases (S1 Fig). However, enzymes involved in lipid independent PA biosynthesis, from Ins(3)P_1_ seem to be complicated and are not well understood [3]. Nevertheless, PA biosynthetic genes encoding other *myo*-inositol enzyme and inositol phosphate kinases are well documented in major plants [12, 13, 20, 21]. Additionally, biochemical and functional analyses of PA biosynthetic genes encoding Ins monophosphate kinase could address the missing steps in the lipid independent pathway.

Genetic studies of LPA mutants have shown that a single recessive gene is responsible for the LPA phenotype in rice and other crop plants [21–24]. The first *lpa* gene encoding inositol 1,3,4-triskisphophate 5/6-kinase (ITPK5/6) was identified in maize, and designated as *Lpa2*. Subsequently, *myo*-inositol kinase gene *Lpa3*, and multidrug resistance protein (MRP) ATP binding cassette (ABC) transporter gene *Lpa1* were identified [12, 13, 25]. In addition, reduction of PA content in *Arabidopsis atipk2β* mutant indicates the inositol 1,4,5-tris-phosphate (IPK2) kinase of lipid dependent pathway is also active the seeds [20]. In rice, several mutants with low seed PA content have been reported [14, 21–23,26–29]. The rice *OsLpa*1 gene has been reported to associated with the reduction in seed PA content and increase in seed P_i_ content, with little change in the total P content in seeds [22, 30]. *OsLpa*1 has been found to share sequence homology with Os09g0572200 (*OsLpa*1 paralog) suggesting possible overlapping or redundant functions [22].

Genetic studies in rice have confirmed that a mutation in the *OsLpa*1 locus generates the LPA phenotype in seeds. Molecular characterization of LPA mutants has previously revealed three alleles of the *OsLpa*1 locus, including KBNT *lpa 1–1*, DR1331-2, and Os-lpa-XQZ-1, responsible for the low PA phenotype of seeds [22, 30]. In the present study, we report a novel allele of *OsLpa*1, *OsLpa1-3*, responsible for a significant reduction in the seed PA content in a new *lpa* rice mutant, Sanggol developed at Kangwon National University, Republic of Korea [31]. Sequence analysis of *OsLpa*1-3 revealed a point mutation in the gene coding sequence. Our data suggest that this mutation is responsible for the LPA phenotype of Sanggol mutant.

## Material and methods

### Plant material

The low PA mutant, Sanggol derived from a *japonica* rice cultivar Ilpum mutagenized with *N*-methyl-*N*-nitrosourea (MNU) [31]. Ilpum was used as the wild type in comparing phenotypic data. Sanggol was crossed with Ilpum to develop F_2_ population. Segregation analysis was performed using the F_2_ population. Both parent cultivars and F_2_ individuals were grown in experimental fields of Seoul National University, Republic of Korea.

### Agronomic trait analysis

To characterize agronomic traits, 15 phenotypic observations were recorded during various stages of plant growth, according to the Standard Evaluation System (SES) for rice, 2014. Yield data was obtained from “3.6 m X 3.6 m” plot size. All agronomic data were analyzed using the Student’s *t*-test in SPSS 16.0 (https://www.ibm.com/analytics/spss-statistics-software) to determine significant differences among low PA mutant, Sanggol, and wild type, Ilpum.

### Analysis of P_i_ and PA content in seeds

Concentrations of P_i_ and PA in seeds were examined using P^31^ nuclear magnetic resonance (P^31^ NMR) spectroscopy [32], with slight modifications.

#### Sample preparation

Fine powdered samples (1 g dry weight) of brown rice were thoroughly mixed with 10 mL of 2.4% HCl in 14 mL Falcon tubes. Samples were incubated at room temperature for 16 h on an HB-201SF shaker (HANBAEK Scientific Co) at 220 rpm, and subsequently centrifuged at 1,500 × *g* (combi 514R, Hanil science Inc.) at 10°C for 20 min. Crude extracts were transferred to a new 14 mL Falcon tube containing 1 g NaCl, and incubated at 25°C for 40 min on a shaker at 220 rpm to dissolve NaCl. Samples were allowed to settle at 4°C for 60 min, and then centrifuged at 1,500 × *g* at 10°C for 20 min.

#### ^31^P NMR

For ^31^P NMR spectroscopy, samples were prepared by mixing 450 μL of NaCl treated acid extract with 450 μl of buffer containing 0.11mM EDTA-disodium salt and 0.75 mM NaOH, 40 mg NaOH, and 100 μL D_2_O in 1.5 mL microtubes. Sample and standard peaks were obtained on a 600 MHz spectrometer using Advance 600 ^31^P NMR system (Bruker, Germany). PA sodium salt and 85% phosphoric acid were used as external standards for peak identification and further analysis [33, 34]. For internal calibration, 1 mM of phenylphosphonic acid was included in 100 μL D_2_O during NMR measurements. All standards were purchased from Sigma-Aldrich, USA.

To determine significant differences in seed PA and P_i_ contents among parents and F_2_ individuals, data were analyzed using the Student’s *t*-test in SPSS 16.0 (https://www.ibm.com/analytics/spss-statistics-software). Additionally, correlation analysis was performed to assess significant association between seed PA (%) and percentage of grain chalkiness (GC) using parents and 20 homozygous F_2_ individuals consisted of mutant, and wild type in SPSS 16.0.

### Expression analysis of PA biosynthetic genes

Genes involved in PA biosynthesis and transport were identified from the RAB-DB and from recent studies [25, 35, 36]. The rice microarray database, RiceX-Pro, shows different expression patterns of most of the PA biosynthetic genes in various tissues and organs [37]. To confirm the expression pattern of PA biosynthetic genes, spikelets were harvested from the wild type Ilpum at 5 days after flowering (DAF), and total RNA was extracted using RNAiso Plus (Takara Bio, Japan). The extracted RNA samples were treated with RNase-free recombinant DNase Ι (Takara Bio, Japan) to eliminate genomic DNA contamination, and first-strand cDNA was synthesized using M-MLV reverse transcriptase (Promega, USA). The fragments of size 200-550bp were amplified from cDNA samples by reverse transcription polymerase chain reaction (RT-PCR) using *myo*-inositol 3-phosphate synthase-1 (*OsMIPS*1), *myo*-inositol 3-phosphate synthase-2 (*OsMIPS*2), *myo*-inositol monophosphate-1 (*OsIMP*1), *myo*-inositol monophosphate-2 (*OsIMP*2), *myo*-inositol kinase (*OsMIK*1), low phytic acid 1 (*OsLpa*1), low phytic acid 1-paralog (*OsLpa*1-P), inositol 1,3,4-triskisphophate 5/6-kinase 1 (*OsITPK*1), inositol 1,3,4-triskisphophate 5/6-kinase 2 (*OsITPK*2), inositol 1,3,4-triskisphophate 5/6-kinase 3 (*OsITPK*3), inositol 1,3,4-triskisphophate 5/6-kinase 4 (*OsITPK*4), inositol 1,3,4-triskisphophate 5/6-kinase 5 (*OsITPK*5), inositol 1,3,4-triskisphophate 5/6-kinase 6 (*OsITPK*6), inositol 1,4,5-tris-phosphate 1 (*OsIpk*1), GLFG lethal 1 (*OsGLE*1), inositol 1,4,5-tris-phosphate 2 (*OsIpk*2), and multidrug resistance protein 13 (*OsMRP13*) genes-specific primers (Table 1) with the following conditions: initial denaturation at 95°C for 2 min, followed by 32 cycles of denaturation at 95°C for 20 s, annealing at 58°C for 40 s, and extension at 72°C for 1 min, and a final extension at 72°C for 5 min. The *Actin* gene was used as an internal control.

**Table 1:** RT-PCR primers used to amplify PA biosynthetic genes.

Gene ID	Gene Name	Primer name	Sequence (5'→3')
Os03g0192700	*OsMIPS*1	*OsMIPS1F*	AGTGGACAAGGTGGTGGTGT
		*OsMIPS1R*	ATCACCACCAATCAGGCAGT
Os10g0369900	*OsMIPS*2	*OsMIPS2F*	GAAGAGCAAGGTGGACAAGG
		*OsMIPS2R*	CATCTTGGTCTGCCCACTCT
Os03g0587000	*OsIMP*1	*OsIMP1F*	GTGGATTTGGTGACGGAGAC
		*OsIMP1R*	ATCGAGACGCAAACAAAAGG
Os02g0169900	*OsIMP*2	*OsIMP2F*	CCTCTTCACACCGCAGGAAT
		*OsIMP2R*	CTGGATGACGCCGAGGAG
Os07g0507300	*OsMIK*1	*OsMIK1F*	TCTACTGGGACGGTGGAGAG
		*OsMIK1R*	TAGCCGCTTCTTGGAGTGAT
Os02g0819400	*OsLpa*1	*OsLPA1F*	TATGTGGGACTAGCGGATGC
		*OsLPA1R*	GAGCAACTGCAACAGGGTCT
Os09g0572200	*OsLpa*1-P	*OsLPA1-P F*	CGGCTGATGTTCCACCTAAT
		*OsLPA1-P R*	TTGACGCTTTCTCAATGTGC
Os10g0103800	*OsITPK*1	*OsITPK1F*	ACAAGGAGTGGCAGCAAGTT
		*OsITPK1R*	CAACCAAGGGCAACGTTAGT
Os03g0230500	*OsITPK2*	*OsITPK2F*	TCTGGTCCTCCAGGAATTTG
		*OsITPK2R*	CCAGTCTTCCACGAAGCTCT
Os03g0726200	*OsITPK*3	*OsITPK3F*	AGGGAGGAACACCCAGAAGT
		*OsITPK3R*	ACCAGAGGCTTTGCCACTAA
Os02g0466400	*OsITPK*4	*OsITPK4F*	ACATGCGCCTCGTCTACC
		*OsITPK4R*	GTTGGAGATGTTGGCGAAG
Os10g0576100	*OsITPK*5	*OsITPK5F*	CCAGCTCCTCAAAGTCTGCT
		*OsITPK5R*	TTTGTCCATGCTCCTTCTCA
Os09g0518700	*OsITPK*6	*OsITPK6F*	GCAAAACGAGGTGCAAGATA
		*OsITPK6R*	GCTTGATTGCATCCCAGAAT
Os04g0661200	*OsIpk*1	*OsIPK1F*	CAACCGGCACCAAACTGTAT
		*OsIPK1R*	CAGAATCAGCTCCAGCATCA
Os02g0596100	*OsGLE*1	*OsGLE1F*	AGACCGCGTCTTGTCTGC
		*OsGLE1R*	GTCGAGCTCGGTGAGGAC
Os02g0523800	*OsIpk*2	*OsIPK2F*	CTCTTCTACAAGCCCCTCCA
		*OsIPK2R*	GAGGCACTTGGCGACGTA
Os03g0142800	*OsMRP*13	*OsMRP13F*	GCTTATTGCATTGGGTAGGG
		*OsMRP13R*	TTACCCGAAGCTCTGATGCT
Os03g0836000	*Actin*	*Actin 4*	AGGCAGTCAGTCAGATCACGA
		*Actin 5*	GAGACATTCAATGCACCAGCA

### Sequence analysis

Genomic DNA and RNA were isolated from young leaves and spikelets of low PA mutant, Sanggol, respectively. cDNA was synthesized with mRNA derived from young leaves and spikelets. Fragments of size 300bp-2000bp were amplified from the coding region and untranslated region (UTR) of 16 PA genes of lipid dependent and independent pathways using gene-specific primers, designed with Primer3 (http://bioinfo.ut.ee/primer3-0.4.0/) (S1 Table). The PCR products were purified using the DNA Purification Kit (Inclone, Korea), and analyzed with an ABI Prism 3730 XL DNA Analyzer (PE Applied Biosystems, USA). In addition, genome sequences of all 16 PA genes in wild type, Ilpum were retrieved from the genome analysis center, National Instrumentation Center of Environmental Management (NICEM), Seoul National University. (http://nature.snu.ac.kr/rice/). Sequence alignment analysis were performed using the Codon Code Aligner software (Codon Code Corporation, USA).

### Candidate gene analysis

To confirm nucleotide polymorphisms in candidate genes, validation primers were designed using Primer3 for cDNA sequencing (Table 2). The PCR products were purified using the DNA Purification Kit (Inclone, Korea), and analyzed with an ABI Prism 3730 XL DNA Analyzer (PE Applied Biosystems, USA). Sequences were aligned using the Codon Code Aligner software (Codon Code Corporation, USA). Simultaneously, BLAST search was performed using the predicted amino acid sequences of the candidate genes in the NCBI database (https://blast.ncbi.nlm.nih.gov/Blast.cgi), and deleterious amino acid substitutions were predicted using Provean web server with proven scores [38].

**Table 2:** Primers used for validating cDNA sequences and genotyping the F_2_ population.

Analysis	Primer name	Sequence (5'→3')	Amplicon size
cDNA validation	*Lpa1-3 F*	GCCATGCCTTCAAGATTAGC	1,186 bp
	*Lpa1-3 R*	TGAAACATTCCCTTGGAACC	
Genotyping	*Lpa1-3-1F*	AGCATTCGCCTGCATGATCG	Homozygous wild type (192 bp), homozygous mutant (174 bp), heterozygous (both 192 bp and 174 bp)
	*Lpa1-3-1R*	CGCTTACCGAACAATGAATG	

### Expression analysis of *Lpa* and lipid dependent PA biosynthesis genes in a low PA mutant, Sanggol and wild type, Ilpum

Total RNA was extracted from leaves at 15 days after germination (DAG) to analyze the expression of *Lpa* and lipid dependent pathway genes, and 5 DAF from spikelets to analyze the expression of *OsLpa*1 in *lpa* mutant, Sanggol and wild type, Ilpum. For the expression analysis of *OsLpa*1 paralog and *OsIpk*2 genes, total RNA was extracted only from spikelets at 5 DAF. RNA extraction was performed as described above, and cDNA was synthesized with mRNA derived from spikelets of 5 DAF. The CDNA was subjected to RT-PCR using gene-specific primers (Table 3). The *Actin* gene was used as an internal control.

**Table 3:** RT-PCR primers used to amplify *Lpa* and *Ipk2* genes.

Gene ID	Transcript	Primer name	Primer sequence (5'→3')	Amplicon size
Os02g0819400	*OsLpa*1.1	*lpa1*.*1 F*	TATGTGGGACTAGCGGATGC	192 bp
		*1pa1*.*1 R*	GAGCAACTGCAACAGGGTCT	
	*OsLpa*1.2	*lpa1*.*2 F*	TATGTGGGACTAGCGGATGC	445 bp
		*lpa1*.*2 R*	GAGCAACTGCAACAGGGTCT	
	*OsLpa*1.3	*lpa1*.*3 F*	ATCTTTCGGGATCAGTGCAT	200 bp
		*lpa1*.*3 R*	TGGCAGCATGTTTCTCCTATC	
Os09g0572200	*OsLpa*1 paralog	*OsLPA2F*	CGGCTGATGTTCCACCTAAT	236 bp
		*OsLPA2R*	TTGACGCTTTCTCAATGTGC	
Os02g0523800	*OsIpk*2-0	*OsIPK2F*	CTCTTCTACAAGCCCCTCCA	291 bp
		*OsIPK2R*	GAGGCACTTGGCGACGTA	
Os03g0836000	*OsActin*	*Actin 4*	AGGCAGTCAGTCAGATCACGA	194 bp
		*Actin 5*	GAGACATTCAATGCACCAGCA	

### Derived cleaved amplified polymorphic sequence (dCAPS) analysis

Genomic DNAs were isolated from all 96 F_2_ plants derived from the cross between Sanggol and Ilpum, and subjected to dCAPS analysis. The F_2_ genotyping primers (Table 2) were designed using dCAPS 2.0 (http://helix.wustl.edu/dcaps/) to validate a single nucleotide substitution (C to T) in the *OsLpa*1 gene in Sanggol, which generates a *Taq*I restriction site (TCGA) in the amplified PCR product. PCR was performed using the following conditions: initial denaturation at 95°C for 2 min, followed by 32 cycles of denaturation at 95°C for 20 s, annealing at 58°C for 40 s, and extension at 72°C for 30 s, and a final extension at 72°C for 1 min. The amplified PCR product was digested with *Taq*I restriction endonuclease (Promega, USA), and separated on 3% agarose gel.

### Multiple sequence alignment and phylogenetic analysis

Amino acid sequences of the Lpa superfamily were obtained from the NCBI protein database (https://blast.ncbi.nlm.nih.gov/Blast.cgi?PAGE=Proteins), and subjected to multiple sequence alignment using the Clustal Omega program (https://www.ebi.ac.uk/Tools/msa/clustalo/). Multiple sequence alignment editing, visualization, and analysis was performed using Jalview 2.10.4 (http://www.jalview.org/). The Lpa and other superfamily proteins obtained from the NCBI protein database were used for phylogenetic analysis. Neighbour-joining tree was constructed using MEGA 7 [39] with 1,000 bootstrap replicates.

### Biocomputational analysis

A three-dimensional (3D) model of Lpa1 protein was produced under the intensive mode of the Phyre2 server [40] (www.sbg.bio.ic.ac.uk/phyre2/html/). The ligand and cofactor were downloaded from the PubChem database (https://pubchem.ncbi.nlm.nih.gov/) for protein ligand analysis. Furthermore, auto docking and 3D model were analyzed using the CLC drug discovery workbench 4.0 (QIAGEN, Denmark). Putative phosphorylation sites were predicted with the GPS 3.0 server (http://gps.biocuckoo.org/) using high cut-off values ranging from 1.36 to 17.72.

## Results

### Agronomic characterization of low PA mutant, Sanggol and wild type, Ilpum

**Fig 1:**
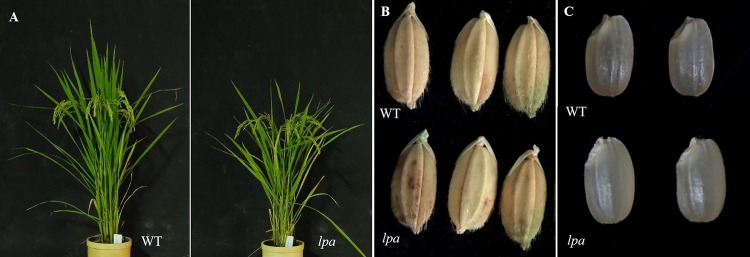
Phenotypic comparison between low PA mutant, Sanggol (*lpa*) and wild type (WT), Ilpum. (A) Whole plant. (B) Spikelet. (C) Mature grain.

Analysis of agronomic traits demonstrated a significant reduction in plant height (cm), number of productive tillers, culm length (cm), the first internodal length (cm), 1,000-grain weight (g), number of spikelets per panicle, number of panicles per plant, and yield components of a low PA mutant, Sanggol compared with a wild type, Ilpum (Table 4 and Fig 1A). By contrast, the number of days to 50% flowering was significantly higher in Sanggol than in Ilpum, indicating delayed flowering in the mutant. In addition, Sanggol exhibited significantly higher percentage of chalky grains compared with the wild type. However, no significant differences were observed between mutant and wild type in morphological characteristics, such as secondary internodal length, grain length, grain width, panicle length, and spikelet fertility (Fig 1B and Fig 1C). Overall, these data indicate that a low PA mutant, Sanggol shows poor agronomic performance with respect to the flowering time, yield, and yield components compared with the wild type, Ilpum.

**Table 4:** Agronomic traits of the wild type, Ilpum and low PA mutant, Sanggol.

Traits^a^	PH (cm)	NPT	DFF	CL (cm)	FIL (cm)	SIL (cm)	GL (mm)	GW (mm)	KGW (g)	GC (%)	NSP	NPP	PL (cm)	SPF (%)	YPP (g)
Wild	97	22	116.7	75	40.5	15	5.01	2.97	20.93	43.62	187	12	22	97	760
Mutant	77	13	122.5	54	35.5	14.83	4.81	3.03	18.9	54.66	165	8	23	96	470
Significance^b^	**	**	**	**	**	NS	NS	NS	**	**	**	*	NS	NS	**

^a^Traits: PH, plant height; NPT, number of productive tillers; DFF, days to 50% flowering; CL, culm length; FIL, first internodal length; SIL, second internodal length; GL, grain length; GW, grain width; KGW, 1,000 grain weight; GC, grain chalkiness; NSP, number of spikelets per panicle; NPP, number of panicles per plant; PL, panicle length; SPF, spikelet fertility; YPP, yield per plot

^b^Asterisks indicate the level of significance (*, *P* < 0.05; **, *P <* 0.01). NS, non-significant.

### Determination of PA and P_i_ content in seeds

**Fig 2:**
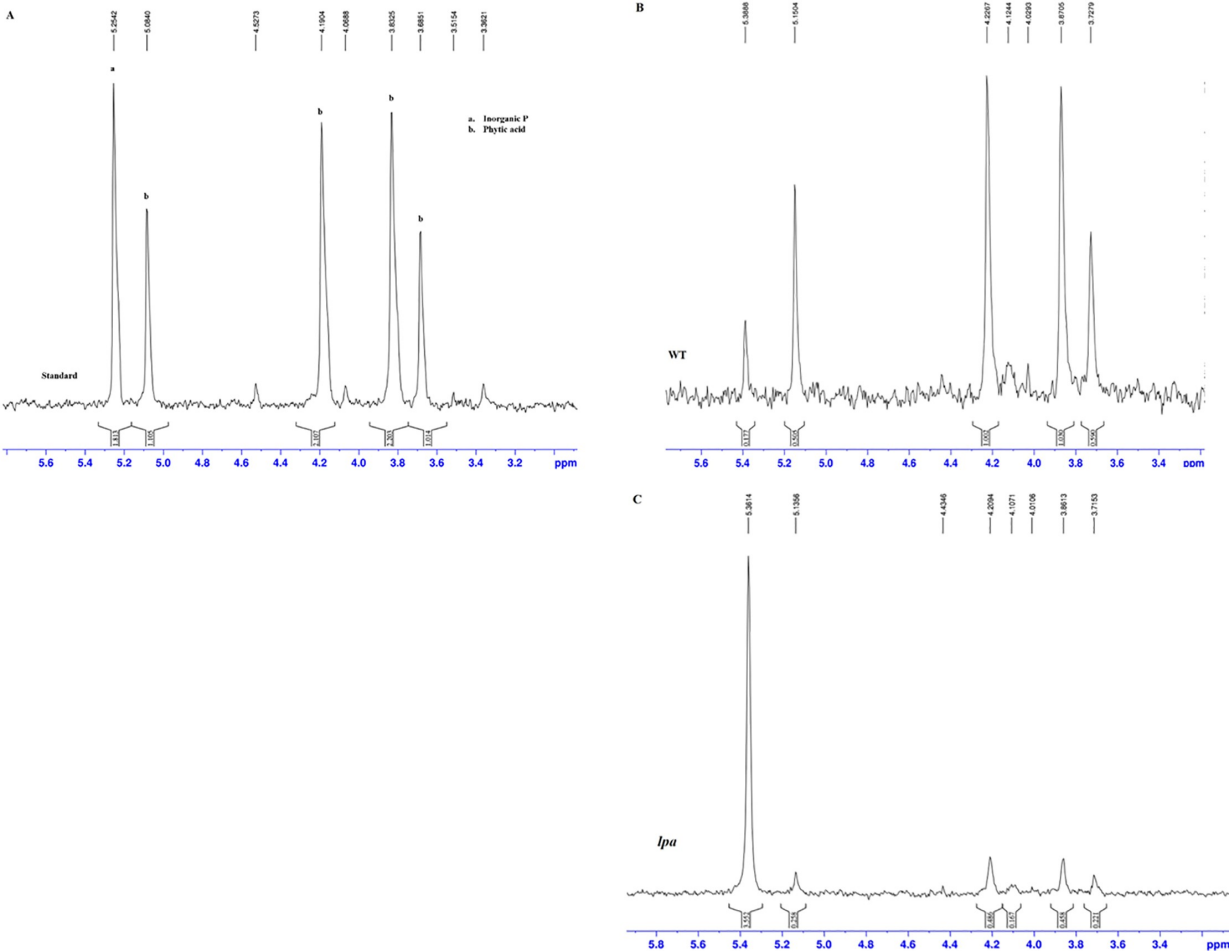
^**31**^**P NMR spectrum of standard, Ilpum (WT), and the mutant, Sanggol (*lpa*)** (A) Reference standard peaks. (B) Wild ‘WT’ (C) *lpa* ‘Mutant’.

To quantify PA and P_i_ content in seeds, brown rice extracts of Sanggol and Ilpum were analyzed via ^31^P NMR spectroscopy. Results showed that PA contents were significantly reduced (49% reduction), and P_i_ content was significantly increased in the seeds of Sanggol compared with Ilpum (Table 5). The ^31^P NMR analysis showed peaks analogous to standard (Fig 2A) for P_i_ and PA peak identification. Similarly, P_i_ and PA analogous peaks were observed for wild type (WT) (Fig 2B), and mutant (*lpa*) (Fig 2C).

**Table 5:** Seed PA and P_i_ content in Sanggol and Ilpum.

Cultivar	PA P (mg g^-1^)	P_i_ (mg g^-1^)	Total P (mg g^-1^)
Ilpum	5.7 ± 0.34	0.1 ± 0.04	5.85 ± 0.34
Sanggol	2.9 ± 0.69*	1.8 ± 0.10**	5.21 ± 0.62

Data represent mean ± standard error (*n* = 3).

Asterisks indicate the level of significance (*, *P* < 0.05; **, *P <* 0.01) between Sanggol and Ilpum.

Additionally, PA and P_i_ amounts were also quantified among 96 F_2_ individuals using ^31^P NMR spectroscopy. Segregation analysis revealed that 77 F_2_ plants showed the wild type phenotype, whereas 19 F_2_ plants showed the mutant phenotype (Table 6), and the phenotype segregation fitted a 3:1 ratio, suggesting that a single recessive allele control the low PA in the seeds of the *lpa* mutant, Sanggol.

**Table 6:** Segregation and co-segregation analysis of seed PA content among 96 F_2_ individuals derived from a cross between low PA mutant, Sanggol and wild type, Ilpum.

Cross	No. of F2 plants	PA phenotype					dCAPS genotyping					
		High PA	Low PA	Expected	χ2	*P*-value†	W*	M*	H*	Expected	χ2	*P*-value†
Sanggol /Ilpum	96	77	19	3:1	1.38	0.23	26	19	51	1:2:1	1.39	0.49

^†^Not significant (*P* > 0.05).

*Wild: homozygous wild type, H: heterozygous, M: homozygous mutant.

Additionally, correlation analysis among PA (%) and GC (%) using parents and 20 homozygous F_2_ individuals revealed that GC had negative significant correlation with PA (r = -0.631**) content in the seeds. Further, homozygous *lpa* individuals in F_2_ population, and *lpa* mutant, Sanggol showed higher GC (%) compared with homozygous wild types in F_2_, and wild type parent, Ilpum. Statistical analysis using Student’s *t*-test revealed significant differences for GC (%) between homozygous wild type and lpa individuals in F_2_ population (S5 Fig).

### Expression of PA biosynthetic gene and sequence analysis

**Fig 3:**
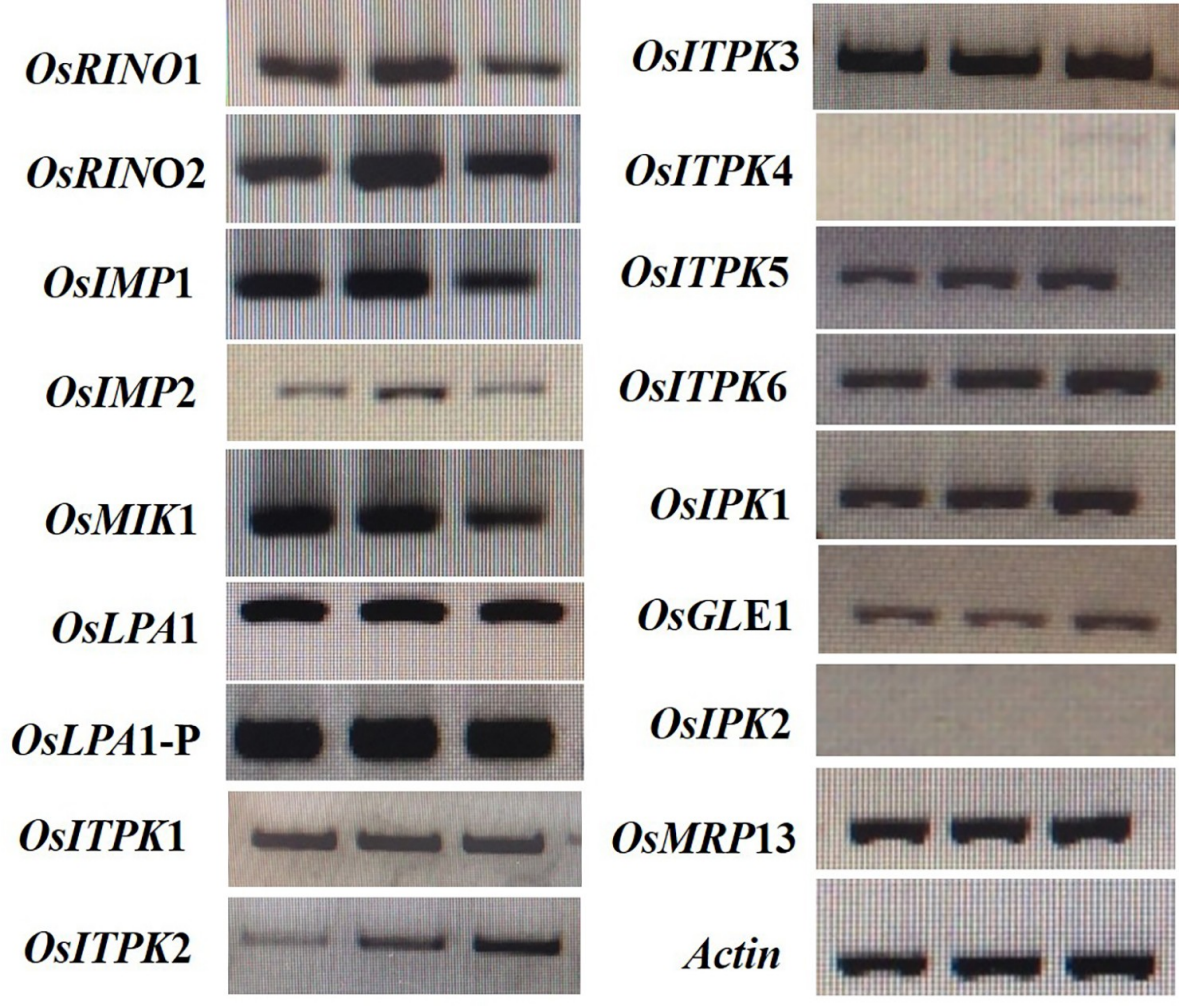
Semi-quantitative RT-PCR analysis of PA biosynthetic genes at 5 DAF in the wild type, Ilpum.

To identify the gene responsible for reduced PA content in seeds, the candidate gene approach was followed. In rice, PA biosynthesis and accumulation begins after flowering [41, 42], and continues until 25 DAF during seed development [43]. Therefore, we extracted total RNA from ‘Ilpum’ spikelets at 5 DAF, and subjected it to RT-PCR analysis. Results showed that 15 genes in the PA biosynthesis pathway were expressed at 5 DAF (Fig 3). Further, we amplified and sequenced 16 genes involved in PA biosynthesis from low PA mutant, Sanggol and wild type, Ilpum (S1 Table).

**Fig 4:**
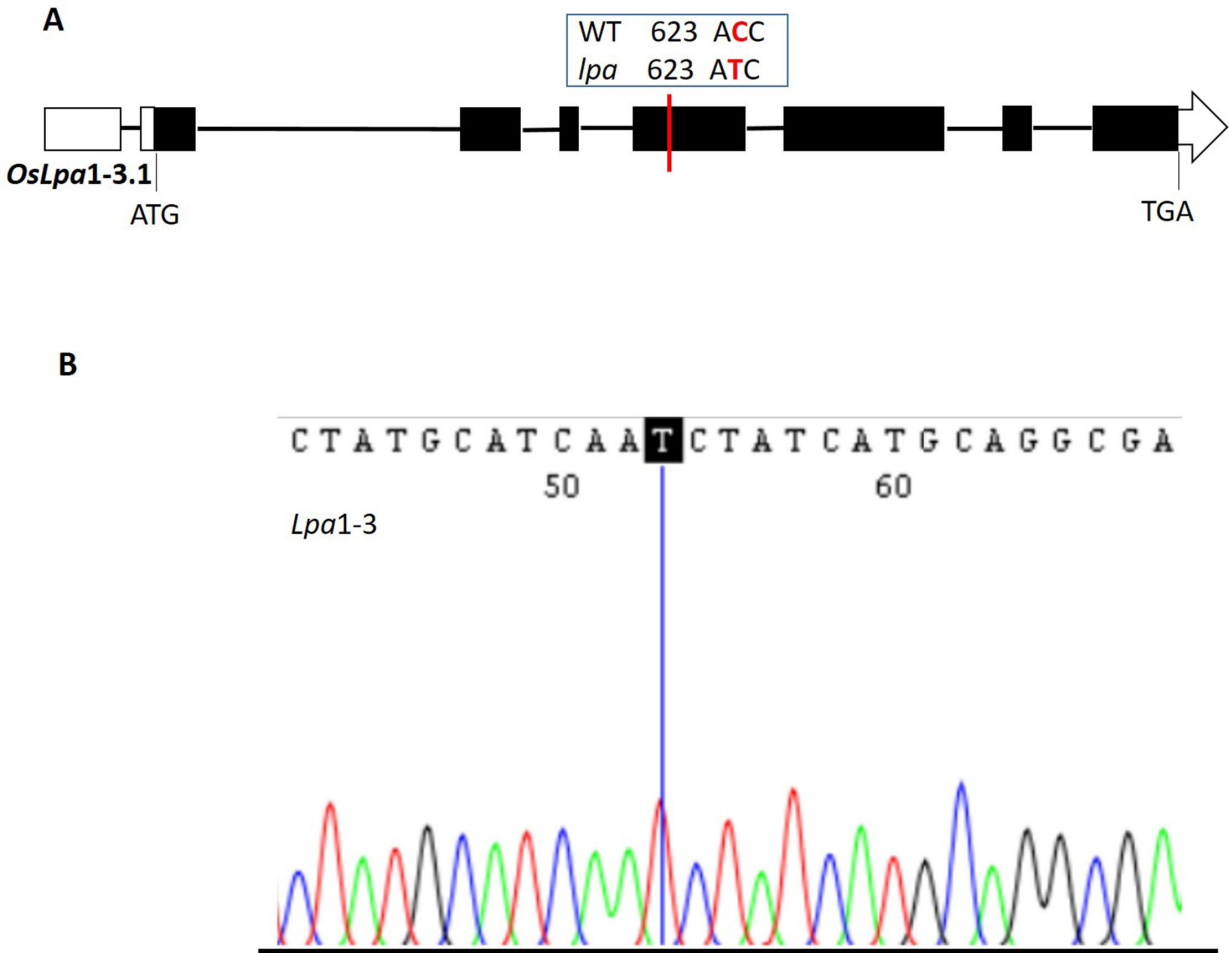
Gene structure of *OsLpa*1-3. (A) Structure of the novel splice variant *OsLpa*1-3.1 carrying the C623T mutation in low PA mutant, Sanggol. Empty boxes represent 5′UTR and 3′UTR, black box represents the coding region, and lines between boxes indicate introns. ATG (start codon) and TGA (stop codon) are shown for each splicing variant. (B) cDNA validation of *OsLpa*1-3.1 showing *Lpa*1-3 allele in the *lpa* mutant, Sanggol.

Sequence analysis of PA biosynthetic genes revealed a single nucleotide polymorphism (SNP) in the *OsLpa*1 gene of *lpa* mutant, Sanggol (Fig 4A); none of the other PA biosynthetic genes showed mutations in *lpa* mutant, Sanggol. Previously, the *OsLpa*1 locus has been mapped to chromosome 2 [11], and narrowed down to a region less than 150 kb using microsatellite and sequence tagged site markers [44]. Further, the *OsLpa*1 has been characterized in *lpa* mutants of rice [22, 30]. The *OsLpa*1 gene encodes three expressed splice variants in rice [22, 35]. Sequence analysis of the *OsLpa*1 locus (position +1 to 2,058 bp; Genbank accession number: MH707666) showed a SNP (C623T) in the fourth exon of the largest splice variant, designated as *OsLpa*1-3.1, in *lpa* mutant, Sanggol. This SNP corresponds to C53T located in the first exon of splice variants of *OsLpa*1-3.2 and *OsLpa*1-3.3 (S2 Fig). Further, sequence analysis of *OsLpa*1-3.1 cDNA confirmed the presence of *lpa1*-3 allele in *lpa* mutant, Sanggol (Fig 4B).

**Fig 5:**
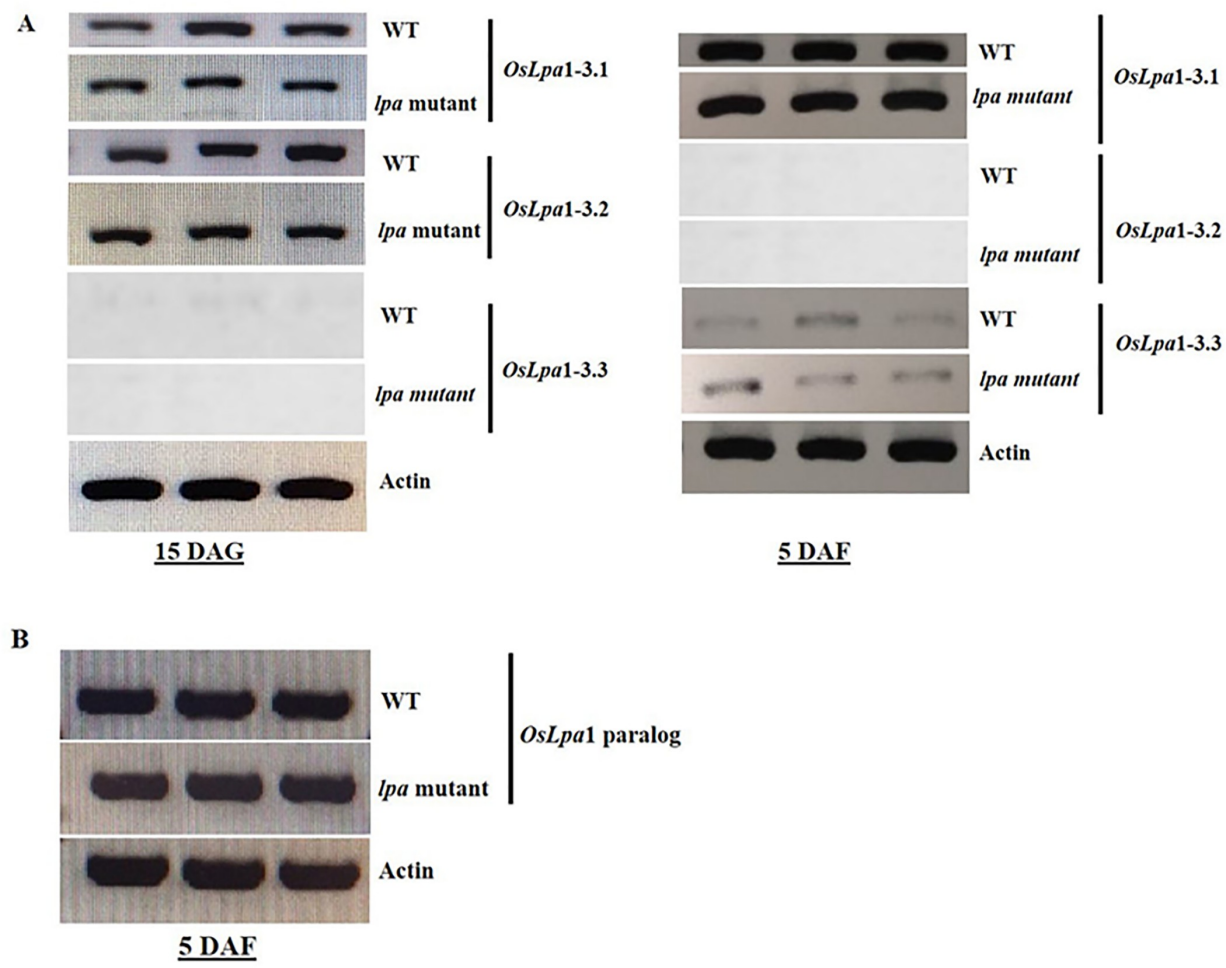
RT-PCR analysis of the *Lpa* gene family in Sanggol and Ilpum. (A) Expression of *OsLpa*1 gene at 15 DAG and 5 DAF. (B) Expression of *OsLpa*1 gene paralog at 5 DAF.

To determine the expression of *OsLpa*1 splice variants in *lpa* mutant and wild type, we performed RT-PCR analysis of *OsLpa*1 gene at 15 DAG using total RNA isolated from leaves and spikelets at 5 DAF. Expression analysis revealed that both *OsLpa*1-3.1 and *OsLpa*1-3.2 were expressed at 15 DAG, with slightly different expression patterns, whereas *OsLpa*1-3.3 showed no expression at 15 DAG in both *lpa* mutant, Sanggol and wild type, Ilpum (Fig 5A), indicating that *OsLpa*1-3.1 and *OsLpa*1-3.2 may play an important role in seedling growth. At 5 DAF, *OsLpa*1-3.1 showed strong expression in both *lpa* mutant, Sanggol and wild type, Ilpum; however, *OsLpa*1-3.3 exhibited low expression in both mutant and wild types, and *OsLpa*1-3.2 exhibited no expression in either types, suggesting *OsLpa*1-3.1 as a candidate transcript responsible for the low PA phenotype of Sanggol mutant. Protein analysis of Lpa1 amino acid sequence predicted deleterious amino acid substitution changes threonine (Thr) to isoleucine (Ile) in *OsLpa*3.1 (Thr208Ile), with a -5.715 proven score. Similarly, deleterious amino acid substitution changes were observed in *OsLpa*3.2 and *OsLpa*3.3 (Thr18Ile), with -5.482 proven scores.

Additionally, existence of the *OsLpa*1 paralog, reported previously by Kim et al. [22], was investigated at 5 DAF in *lpa* mutant, Sanggol and wild type using RT-PCR. The *OsLpa*1 paralog exhibited strong expression in both *lpa* mutant, Sanggol and wild type (Fig 5B), suggesting that sequence variation in the coding region of *OsLpa*1 was responsible for the low PA content of Sanggol seeds. In addition, reduction of PA content in *Arabidopsis atipk2β* mutant indicates the IPK2 kinase of lipid dependent pathway is active the seeds [20]. We also ruled out the possibility for seed PA biosynthesis similar to *Arabidopsis* in low PA mutant, Sanggol. However, our RT-PCR results showed no expression of *OsIpk*2, a key PA biosynthesis gene in the lipid dependent pathway (data not shown), suggesting that the lipid dependent pathway is not active in low PA mutant, Sanggol or wild type, Ilpum.

**Fig 6:**
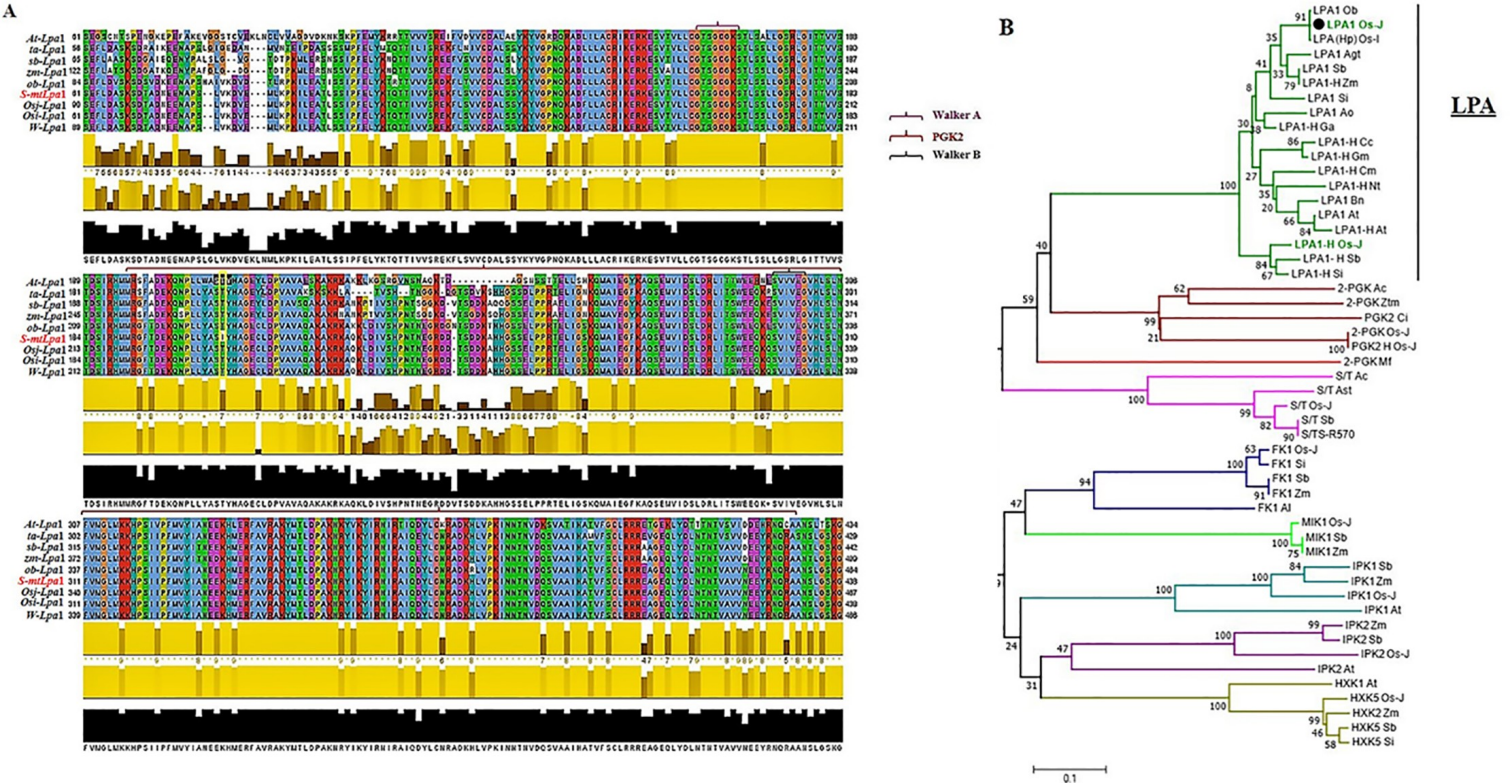
Multiple sequence alignment and phylogenetic analysis of Lpa protein. (A) Multiple sequence alignment of Lpa1 protein from Sanggol (s-mtLpa1) and other major plant species. The P-loop kinase domain of 2-PGK found in *Methanothermus fervidus* is marked with a red line. P-loop is an ATP/GTP binding site motifs (Walker A and Walker B) are indicated using pink and black arrowheads, respectively. The yellow box shows a single amino acid substitution in the conserved kinase domain in *lpa* mutant, Sanggol. (B) Phylogenetic relationship among protein families of various kinases, including 2-PGK, serine threonine protein kinase (S/T), fructokinase (FK), *myo*- inositol kinase 1 (MIK), inositol 1,3,4,5,6 penta*kis*phosphate 2-kinase (IPK1), inositol 1,4,5-tris-phosphate kinase (IPK2), hexokinase 1 (HXK), and hexokinase 2, with the Lpa protein clade. Phylogenetic tree was constructed using amino acid sequences of Lpa and other kinase protein families from selected species using the neighbour-joining method with 1,000 bootstrap replicates. Protein homologs are indicated with an ‘H’. ob, *Oryza brachyantha*; Os-J, *Oryza sativa* L. *japonica*; Osi, *Oryza sativa* L. *indica*; Agt/Ast, *Aegilops tauschii*; sb, *Sorghum bicolor*; zm, *Zea Mays*; Si, *Setaria italica*; Ao, *Asparagus officinalis*; Ga, *Gossypium arboretum*; Cc, *Cajanus cajan*; Gm, *Glycine max*; Cm, *Cucurbita maxima*; ta, *Nicotiana tabacum*; Bn, *Brassica napus*; At, *Arabidopsis thaliana*; Ac, *Ananas cosmos*; Ztm, *Zotera marina*; Ci, *Chrysanthemum indicum*; Mf, *methanothermus fervidus*; R-570, *Saccharum*; Al, *Arabidopsis lyrata*.

Next, we performed multiple sequence alignment of Lpa1 amino acid sequences of Sanggol and other major plant species. Results revealed an amino acid substitution in the conserved kinase domain in Sanggol (Fig 6A), thus showing the impact of a SNP in gene coding sequence. The kinase domain of Lpa1 shows weak homology with that of 2-phosphoglycerate kinase (2-PGK) found in hyperthermophilic methanogens [22]. However, there is structural similarity among the substrates and products of 2-PGK and Lpa1 [45].

Phylogenetic analysis revealed a strong relationship among the kinase proteins in the glycolysis and PA biosynthesis pathways. In contrast, Lpa proteins are clustered with other 2-phosphoglycerate kinases 92-PGK) proteins (Fig 6B).

### Co-segregation analysis of low PA phenotype with *lpa1-3* allele

**Fig 7:**
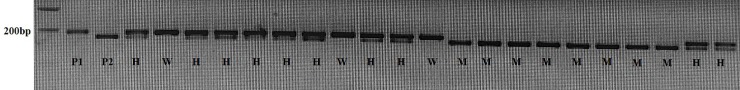
dCAPS analysis of *lp1*-3 allele in the F_2_ population derived from a cross between Sanggol and Ilpum. P1, ‘Ilpum’; P2, ‘Sanggol’; W, homozygous wild type; M, homozygous mutant; H, heterozygous.

A dCAPS assay was applied to determine the co-segregation of *lpa*1-3 allele with the low PA phenotype (Fig 7). A pair of dCAPS markers amplified a 192 bp PCR product. Digestion of this PCR product with *Taq*I yielded a 174 bp fragment in Sanggol, but an uncut fragment (192 bp) in Ilpum. Genotyping the F_2_ individuals using this dCAPS marker showed a segregation ratio, which was consistent with the expected ratio of 1:2:1 (Table 3). In addition, the dCAPS marker genotype co-segregated with the low PA phenotype in the F_2_ population. Statistical analysis using Student’s *t*-test revealed significant differences in the seed PA (S3 Fig) and P_i_ (S4 Fig) contents of Ilpum, Sanggol, and F_2_ individuals.

### Biocomputational analysis

**Fig 8:**
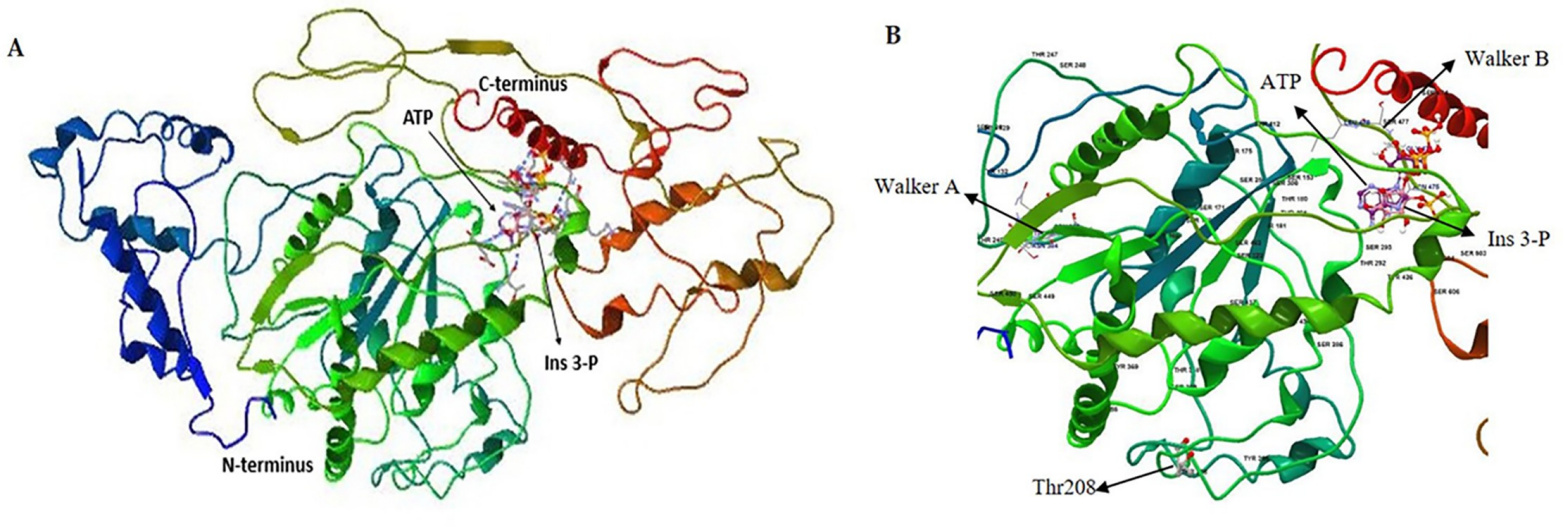
Three-dimensional (3D) model of Lpa1 protein structure. (A) 3D model of Lpa1 protein showing Ins(3)P_1_ (ligand) binding site and ATP (cofactor). (B) Thr208Ile substitution is indicated with an arrow, and the P loop containing Walker A and Walker B motifs is shown.

Structural analysis of Lpa1 using molecular docking of ligand and cofactors showed that Ins(3)P_1_ could bind to the active site of Lpa1 protein, with ATP as a cofactor for catalysis (Fig 8A). Detailed view of the 3D protein model showed that Thr residue at amino acid position 208 was located in the kinase loop (Fig 8B) on the outer surface of the protein, adjacent to the entry site of the binding pocket, thus indicating its potential involvement in regulating the enzyme activity of Lpa1 protein. In addition, GPS 3.0 predicted Thr208 residue as a putative phosphorylation site, with a score of 9.66 above the cut-off value of 8.31. In previous studies, biochemical characterization of the regulatory mechanisms of various other metabolic enzymes has shown that amino acid substitutions are responsible for the reduction in enzyme activity of mutant proteins compared with wild type proteins [46, 47]. Altogether, our results suggest that Thr208Ile amino acid substitution regulates the enzyme activity of Lpa1 protein via phosphorylation in *lpa* mutant, Sanggol.

## Discussion

To date, several genes controlling PA biosynthesis have been reported in major crop plants [13, 20, 25, 28, 48–50]. The biosynthesis of PA proceeds via two major routes: a lipid dependent pathway, which operates in all plant tissues, and lipid independent pathway, which operates predominantly in seeds [3, 17]. In rice, molecular characterization of genes encoding MIPS, MIK, Lpa1, ITPK5/6, and IPK1 has revealed association with the low PA phenotype [21–25]. The first step of PA biosynthesis involves the conversion of D-glucose-6-phosphate to Ins(3)P_1_ by MIPS [19], which is followed by a series of phosphorylation steps, leading to the formation of InsP_6_ (S1 Fig). However, biochemical pathways leading to the conversion of Ins(3)P_1_ to InsP_4_, and the enzymes involved are very complex and not yet fully understood in plants [3].

Understanding the genetic basis of low PA phenotype is important for developing cultivars with low PA content in seeds. Therefore, we obtained the low PA mutant, ‘Sanggol’ from Kangwon National University, Republic of Korea [31, 46]. In this study, Sanggol showed relatively poor agronomic performance compared with the wild type, Ilpum (Table 4). These results are in agreement with previous studies showing superior agronomic performance of wild type compared with the low PA mutants [5, 14]. Edwards et al. [51] report an association between *Lpa*1 locus and grain chalkiness in rice. Similarly, GC (%) had negative significant correlation with PA, indicating that the low PA phenotype interacts with grain chalkiness. Thus, results of this study and previous studies suggest that the *lpa* allele plays an important role in determining the yield potential and seed quality of rice.

Phenotypic analysis using P^31^ NMR spectroscopy showed a significant reduction in PA content and an increase in P_i_ content in Sanggol seeds (Table 5). Expression analysis of PA biosynthetic genes in spikelets of the wild type Ilpum at 5 DAF indicated that 15 genes from the lipid independent pathway were possibly responsible for the low PA content in Sanggol (Fig 3). Our data showed that a point mutation in the *OsLpa*1 locus was associated with low PA content in Sanggol seeds. Previous studies have also shown that rice low PA mutants exhibit a reduction in seed PA content because of SNPs [25, 30]. Candidate gene sequencing (Fig 4) and co-segregation analysis (Fig 7 and Table 6) confirmed that a new single recessive allele of *Lpa1*, designated as *lpa1-3*, was responsible for the low PA phenotype of *lpa* mutant, Sanggol because of a C/T SNP located at nucleotide position 623 in *OsLpa*1, resulting in a single amino acid substitution (Thr208Ile). In a previous study, the *japonica* mutant ‘KBNT *lpa1-1*’ exhibited a 28% reduction in seed PA content because of a SNP (C/G to T/A), resulting in a nonsense mutation at amino acid position 409 whereas the DR1331-2 (*lpa1-2)* mutant showed a 48% reduction in seed PA content because of a single nucleotide deletion (T/A) at position 313, causing a frame shift mutation [22]. In addition, molecular characterization of the *indica* mutant ‘Os-lpa-XQZ-1’ shows the deletion of a 1,475 bp fragment in *lpa1-1*, resulting in a 38% reduction in seed PA content [30].

The *OsLpa*1 gene encodes three splice variants, all of which are expressed in seeds, suggesting that these variants play different roles in rice seed development [22, 35]. However, RT-PCR analysis of *OsLpa*1 locus revealed that *OsLpa*1-3.1 expression exhibited both vegetative and seed specificity, which indicates a major role of *OsLpa*1-3.1 in PA biosynthesis; however, *OsLpa*1-3.2 and *OsLpa*1-3.3 showed significant and dynamic changes at 15 DAG and 5 DAF, respectively (Fig 5A), suggesting that these variants may play important roles in seedling growth and seed development, respectively. This finding is consistent with a previous study in rice [52]. Additionally, we investigated the expression of *OsLpa*1 paralog on chromosome 9 (Os09g0572200), that encodes a protein homology to *OsLpa*1-3 and a IPK2 kinase is specific for the lipid dependent pathway, *OsIpk*2 (Os02g0523800), in spikelets at 5 DAF, to provide an alternative explanation for the low level of PA in *lpa* mutant seeds. However, expression analysis *OsLpa*1 paralog gene suggests possible overlapping functions for PA biosynthesis in *lpa* mutant, Sanggol (Fig 5B).

According to a previous study, *OsLpa*1 shows a weak homology to P-loop kinase domain of 2-PGK found in *Methanothermus fervidus* [22]. 2,3-bisphosphoglycerate (2,3-BPG), derived from 2-PGK, is a strong inhibitor of inositol polyphosphate 5-phosphatases [53]; thus, removing this inhibition may degrade inositol polyphosphate intermediates, causing a reduction in seed PA content in low PA mutants [22, 54]. Based on the structural similarity among substrates and products of *OsLpa*1 and 2-PGK, it is possible that Lpa1 protein functions as a kinase [3]. Further, Sequence analysis search of *OsLpa*1-3 showed that P-loop NTPase domain super-family is characterized by two ATP/GTP binding motifs, i.e. Walker A [GxxxxGK(S/T)] and Walker B [hhhhEG] thus, indicated *OsLpa*1-3.1 has both Walker A and Walker B motifs, *OsLpa*1-3.2 and *OsLpa*1-3.3 doesn’t contain Walker A motif and mutation residue (Thr18) located in N-terminal, suggesting *OsLpa*1-3.2 and *OsLpa*1-3.3 could be non-functional proteins. However, existence and possible involvement of *OsLpa*1-3.2 and *OsLpa*1-3.3 in PA biosynthesis remains to be determined.

Additionally, our results revealed a single amino acid substitution (Thr208Ile) in the kinase domain of *Lpa1* in Sanggol. It is possible that the Lpa1 encodes an Ins(3)P_1_ kinase, which is phylogenetically clustered separately with other kinases in glycolysis and PA biosynthesis. From the molecular docking analysis, it is evident that Ins(3)P_1_ binds to the Lpa1 protein, with ATP as a cofactor for catalysis (Fig 8A). Overall, these results suggest that Lpa1 protein functions as a kinase, and is probably involved in the conversion of Ins(3)P_1_ to *myo*-inositol 3,4-bisphosphate [Ins (3,4) P_2_].

In *Arabidopsis*, aspartic acid (Asp) to alanine (Ala) substitutions at amino acid positions 98 and 100 (Asp98Ala and Asp100Ala) in two genes encoding inositol polyphosphate kinases result in inactive enzymes and LPA phenotypes [55]. Similarly, analysis of phosphorylation deficient mutants in yeast and human shows decreased MIPS activity compared with wild type because of amino acid substitutions at phosphorylation sites [48]. Several studies of various kinases and other metabolic enzymes show reduced enzyme activity of the mutant protein because of Thr and other amino acid substitutions at phosphorylation sites [47, 56–60]. Therefore, we speculate that a point mutation (C623T) causing Thr208Ile amino acid substitution in the loop adjacent to the entry site of the binding pocket of *OsLpa*1 is responsible for the altered enzyme activity of *OsLpa*1-3.1, resulting in reduced PA biosynthesis in Sanggol mutant seeds. Additionally, enzyme activity analysis is necessary to confirm the association of Thr208Ile substitution with the reduction in seed PA content in low PA mutant, Sanggol.

Though *lpa* rice lines are accompanied with negative impact on plant performance, studies in barley and soyabean showed that *lpa* mutants, barley *lpa* 1–1, and soyabean *Gm-lpa-ZC-2*, have not associated with any yield reduction [49, 61]. These results suggest the possibility of developing *lpa* lines when much attention is given simultaneously to both yield performance and low PA, particularly through the modulation of metabolic cross talk between P and grain yield. The results of Sanggol mutant reported in this study will facilitate the development of low PA lines with increased seed micronutrient bioavailability, high P uptake, and enhanced agronomic performance, through marker assisted introgression of the *lpa1-3* allele into high yielding elite rice varieties.

## Supporting information

S1 Fig Schematic representation of the phytic acid (PA) biosynthetic pathway. Glu6p, glucose-6-phosphate; Ins, *myo*-inositol; PtdIns, phosphatidyl inositol; MIPS, *myo*-inositol-3-phosphate synthase; IMP, *myo*-inositol monophosphatase; MIK, *myo*-inositol kinase; LPA1, low phytic acid 1; ITPK, inositol 1,3,4-triphosphate 5/6-kinase; IPK1, inositol 1,3,4,5,6 penta*kis*phosphate 2-kinase; PIS, phosphatidyl inositol phosphate synthase; PI4K, phosphatidyl inositol 4-kinase; PIP5K, phosphatidyl inositol 4 phosphate 5-kinase; PLC, phospholipase C; IPK2, inositol 1,4,5-tris-phosphate kinase; MRP, multidrug resistance protein.
(TIF)

S2 Fig Gene structure of *OsLpa*1-3.2 and *OsLpa*1-3.3 splice variants. Mutations (C53T) in the first exon of *OsLpa*1-3.2 and *OsLpa*1-3.3 are indicated with red lines. Empty boxes represent 5′ and 3′ untranslated regions (UTRs), black box represents the coding region, and lines between boxes indicate introns. ATG (start codon) and TGA (stop codon) are shown.
(TIF)

S3 Fig Statistical analysis of seed PA content among F_2_ plants derived from a cross between the *lpa* mutant, Sanggol and wild type, Ilpum. Data were analyzed using the Student’s *t*-test. M, mutant; W, wild type.
(TIF)

S4 Fig Statistical analysis of inorganic phosphorous (P_i_) content in seeds of F_2_ plants. Data were analyzed using the Student’s *t*-test. M, mutant; W, wild type.
(TIF)

S5 Fig Statistical analysis of GC (%) in seeds of F_2_ plants. Data were analyzed using the Student’s *t*-test. M, mutant; W, wild type.
(TIF)

S1 Table Primers used to sequence 16 PA biosynthetic genes in the lipid dependent and independent pathways. (DOCX)
