# Supplementary figures and images for: Novel allelic variant of Lpa1 gene associated with a significant reduction in seed phytic acid content in rice (Oryza sativa L.)
Source: PLoS One. 2019 Mar 14;14(3):e0209636. doi: 10.1371/journal.pone.0209636 (PMC6417671; doi:10.1371/journal.pone.0209636)

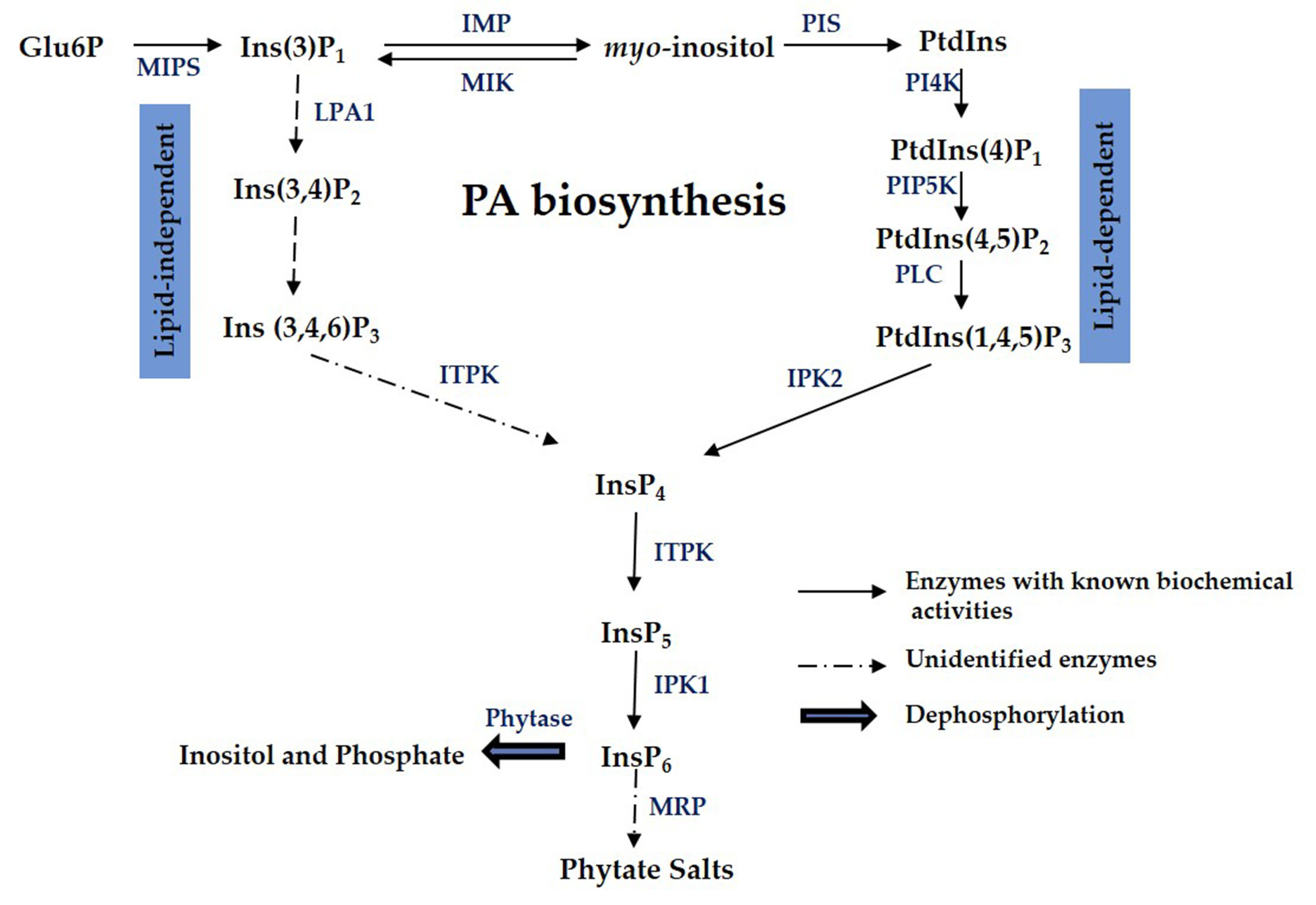

Supplement: S1 Fig — Glu6p, glucose-6-phosphate; Ins, myo-inositol; PtdIns, phosphatidyl inositol; MIPS, myo-inositol-3-phosphate synthase; IMP, myo-inositol monophosphatase; MIK, myo-inositol kinase; LPA1, low phytic acid 1; ITPK, inositol 1,3,4-triphosphate 5/6-kinase; IPK1, inositol 1,3,4,5,6 pentakisphosphate 2-kinase; PIS, phosphatidyl inositol phosphate synthase; PI4K, phosphatidyl inositol 4-kinase; PIP5K, phosphatidyl inositol 4 phosphate 5-kinase; PLC, phospholipase C; IPK2, inositol 1,4,5-tris-phosphate kinase; MRP, multidrug resistance protein. (TIF) [file pone.0209636.s001.tif]

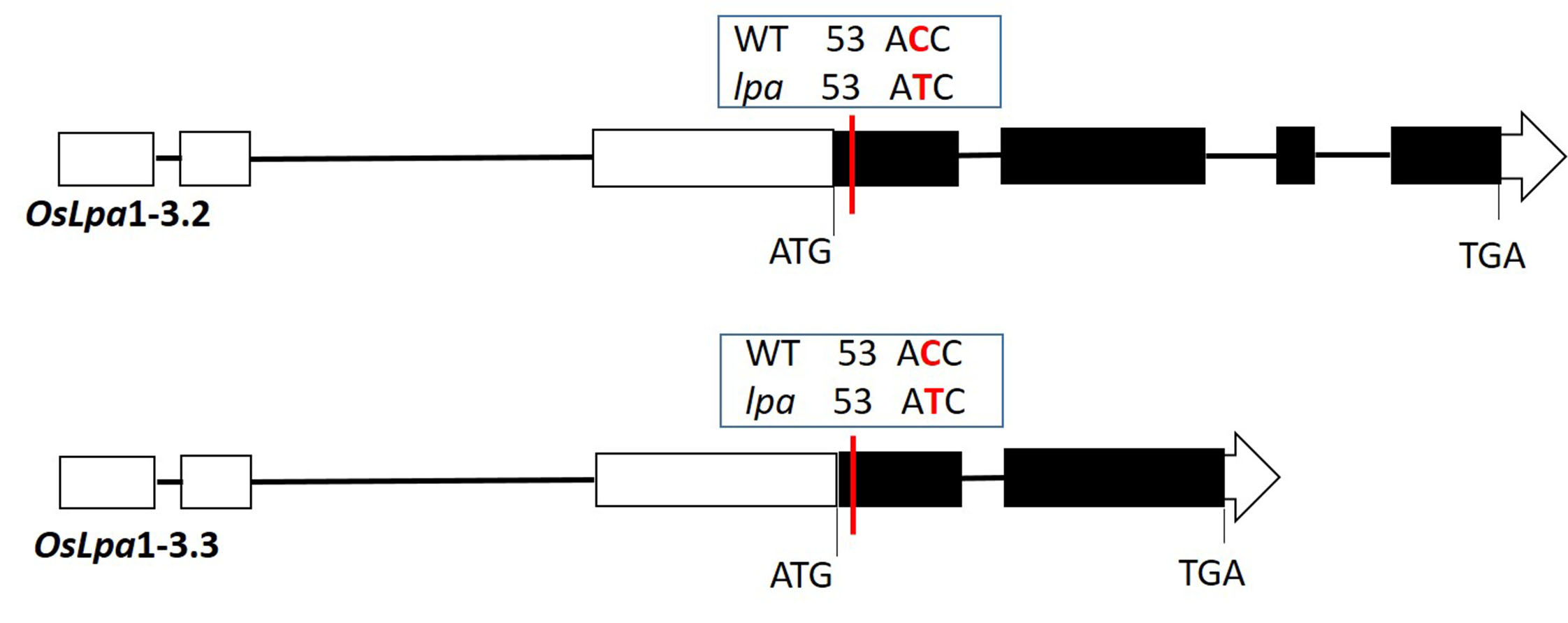

Supplement: S2 Fig — Mutations (C53T) in the first exon of OsLpa1-3.2 and OsLpa1-3.3 are indicated with red lines. Empty boxes represent 5′ and 3′ untranslated regions (UTRs), black box represents the coding region, and lines between boxes indicate introns. ATG (start codon) and TGA (stop codon) are shown. (TIF) [file pone.0209636.s002.tif]

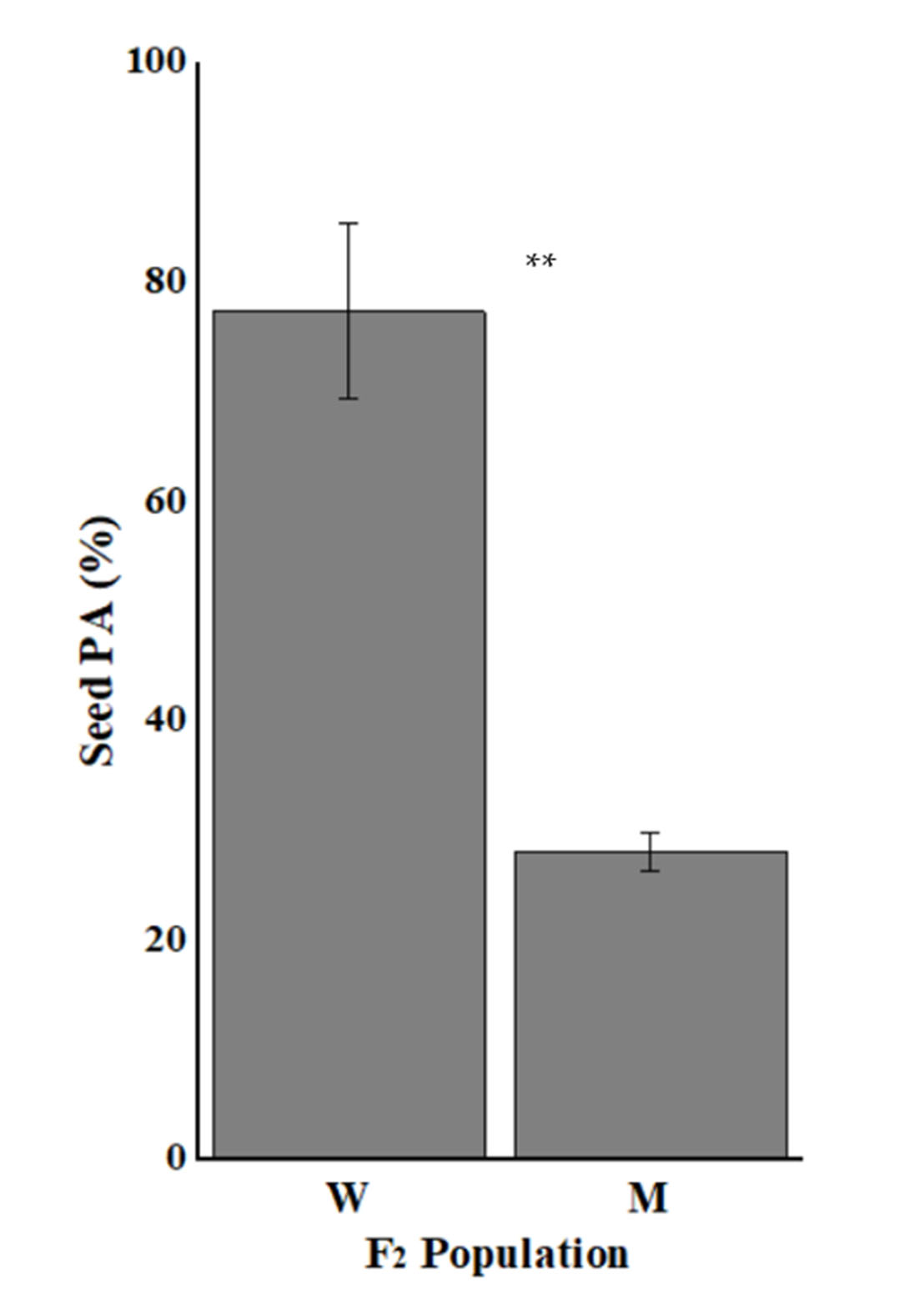

Supplement: S3 Fig — Data were analyzed using the Student’s t-test. M, mutant; W, wild type. (TIF) [file pone.0209636.s003.tif]

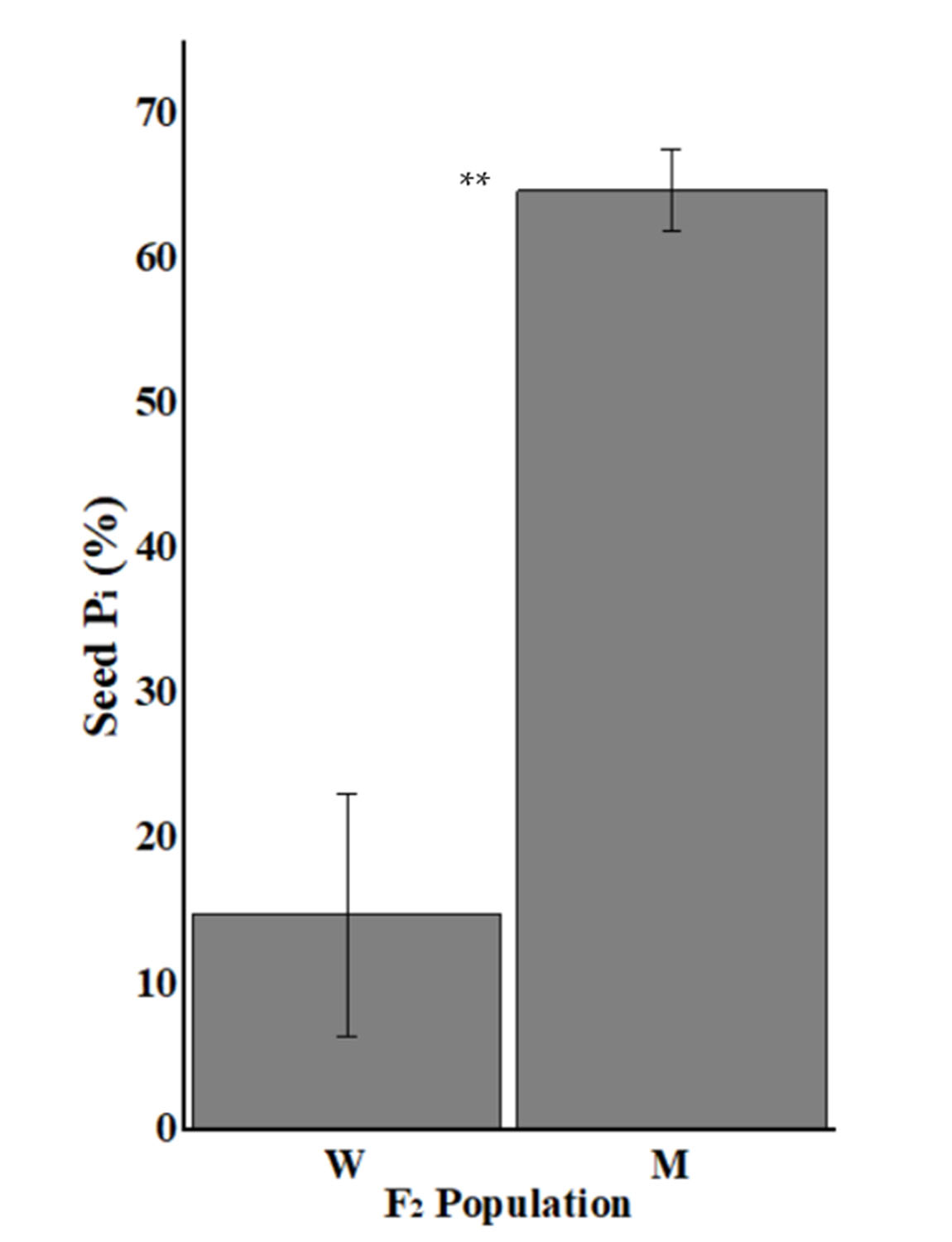

Supplement: S4 Fig — Data were analyzed using the Student’s t-test. M, mutant; W, wild type. (TIF) [file pone.0209636.s004.tif]

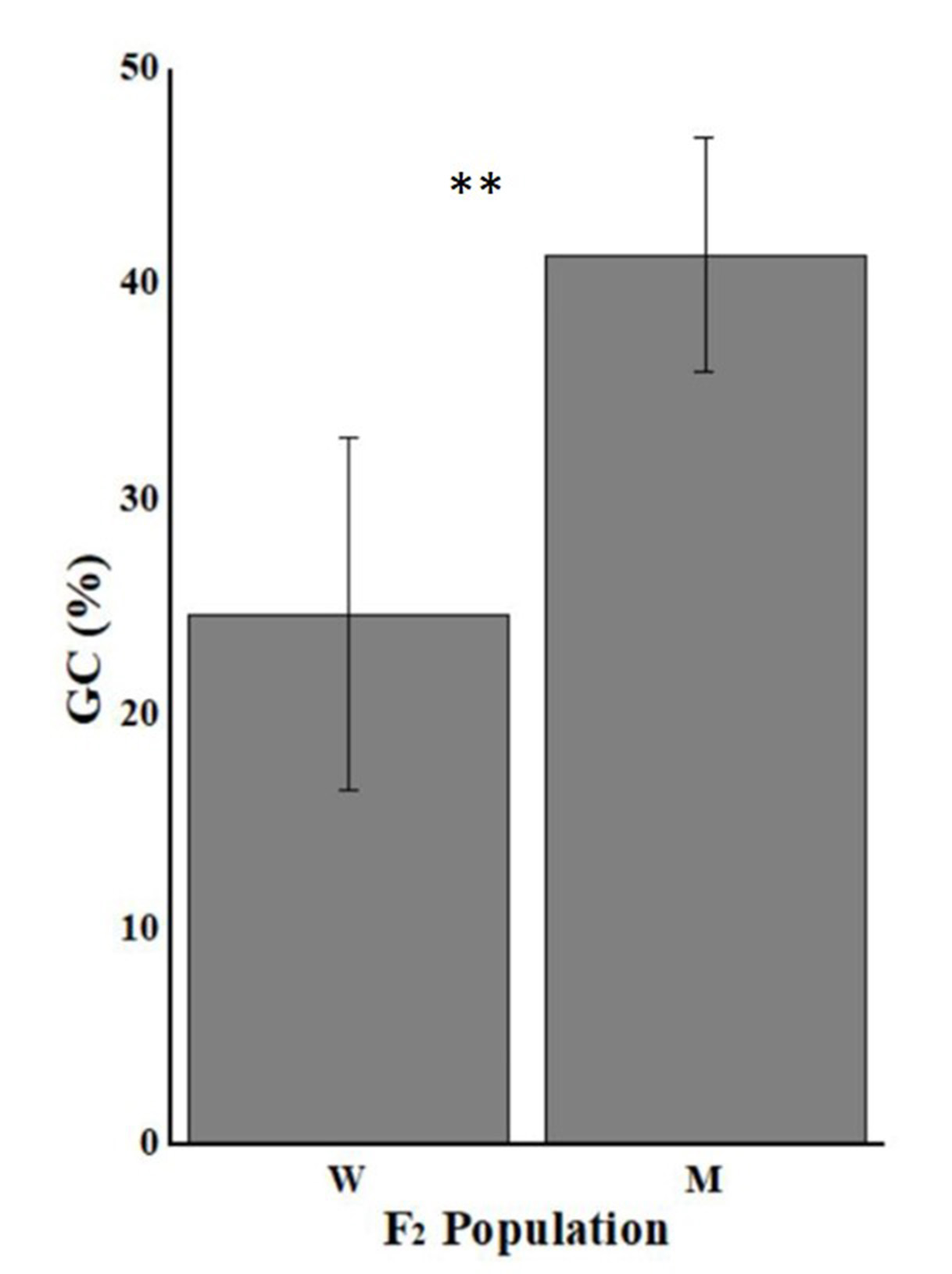

Supplement: S5 Fig — Data were analyzed using the Student’s t-test. M, mutant; W, wild type. (TIF) [file pone.0209636.s005.tif]
