# Supplementary material for: Novel allelic variant of Lpa1 gene associated with a significant reduction in seed phytic acid content in rice (Oryza sativa L.)
Source: PLoS One. 2019 Mar 14;14(3):e0209636. doi: 10.1371/journal.pone.0209636 (PMC6417671; doi:10.1371/journal.pone.0209636)
Supplement: S1 Table — (DOCX) [file pone.0209636.s006.docx]

**S1 Table.**

| **Gene ID** | **Primer name** | **Sequence (5'-3')** |
| --- | --- | --- |
| Os03g0192700 | *OsRINO*1-1F | GGACGAACTTGCAAACACAG |
|  | *OsRINO*1-1R | AATGTCGTTTTGAGCAGACG |
|  | *OsRINO*1-2F | CGTTTGGTCTCCGTGTTTTT |
|  | *OsRINO*1-2R | ACAAAGGTGACACACGATGG |
|  | *OsRINO*1-3F | AGCTACAACGGGGAGGAGAT |
|  | *OsRINO*1-3R | GAGGTGGGCTACGAAAACAA |
|  | *OsRINO*1-4F | TGGTGATGATTTCAAGAGTGG |
|  | *OsRINO*1-4R | TGCAGTTCTGCATAAACCATC |
| Os10g0369900 | *OsRINO*2-1F | CAGGCAACCAAACACACCTATATTT |
|  | *OsRINO*2-1R | TGCAATGAACTCATGCATTTTTGTG |
|  | *OsRINO*2-2F | CGTCTCCATGGATCCTGATT |
|  | *OsRINO*2-2R | GTTCGACCTGCTCCTTCTTG |
|  | *OsRINO*2-3F | AAGAAGGAGCAGGTCGAACA |
|  | *OsRINO*2-3R | CCCGAGTTGGAGTTCATCAT |
|  | *OsRINO*2-4F | TACATATGATGAACTCCAACTCGGG |
|  | *OsRINO*2-4R | TAAAGTTAACAGGCCAAAAGTGCAT |
|  | *OsRINO*2-5F | CCAAACCAAACACGTTACACATCTA |
|  | *OsRINO*2-5R | ACAGCATATGCAGATGTTGAAGAAG |
| Os03g0587000 | *OsIMP*1-1F | ACCGTTTGGTTTATCGGTCA |
|  | *OsIMP*1-1R | GAAAGACAGTAGCGGCCAAA |
|  | *OsIMP*1-2F | GAGGTAAGTGATACCATCAAGAGTCA |
|  | *OsIMP*1-2R | CGAATTTGGACATGCTACCTT |
| Os02g0169900 | *OsIMP*2-1F | CTCATGGGAGCGAATTTAGG |
|  | *OsIMP*2-1R | GGGAGGACGGAGAAGATACC |
|  | *OsIMP*2-2F | ATGGCACGGTATCTTCTCC |
|  | *OsIMP*2-2R | TTAAAAGTCGGTAGGGTATTTGTC |
|  | *OsIMP*2-3F | ACCCGACAAATACCCTACCG |
|  | *OsIMP*2-3R | GAATTTCAGCCTAATTTGGTGTCCA |
| Os07g0507300 | *OsMIK*1-1F | AGCTGCCAACTAGGAGCAGT |
|  | *OsMIK*1-1R | TGAAAACCTGAAACGCATTG |
|  | *OsMIK*1-2F | GAACTTCAGGTAGCTCTTGTCG |
|  | *OsMIK*1-2R | TAGCCGCTTCTTGGAGTGAT |
|  | *OsMIK*1-3F | GCCTGGCATAGCTGTGTTTA |
|  | *OsMIK*1-3R | GTAGCCTTATGCGGAAGTGG |

| **Gene (RAP-ID)** | **Primer Name** | **Sequence (5'-3')** |
| --- | --- | --- |
| Os02g0819400 | *OsLPA*1-1F | CTAATCCAAAGGGTGGAGCA |
|  | *OsLPA*1-1R | GCGGAAGAATCACGAGAGAG |
|  | *OsLPA*1-2F | TCCCGCTTAATTTCTGCCTA |
|  | *OsLPA*1-2R | ACGATGGTTGTTTGGGTCTT |
|  | *OsLPA*1-3F | GCAAGAGGGTGTTCAATGTG |
|  | *OsLPA*1-3R | TGTCGTCTCTGCCTTCATTG |
|  | *OsLPA*1-4F | GGAAGGCACAGAAGCTTGAC |
|  | *OsLPA*1-4R | GGCAATCTGACACCTTTGGT |
|  | *OsLPA*1-5F | CTTGAGAGCTGATGGCATTG |
|  | *OsLPA*1-5R | ACCACCATACCCACGAAGAT |
| Os09g0572200 | *OsLPA*2-1F | CCGTGTCAAAGGACTCCAAT |
|  | *OsLPA*2-1R | ACTGTGCTGTGCTGCTCAAG |
|  | *OsLPA*2-2F | CTCTTTCCACCTTGCTGGTT |
|  | *OsLPA*2-2R | TCAGCCGCTTCTTCTTCTTC |
|  | *OsLPA*2-3F | TTGGATGGACATTGTAAAGAGG |
|  | *OsLPA*2-3R | CAATCTTCATCGACGGAACC |
|  | *OsLPA*2-4F | CCACACTAATTTGAGGATCTGAAG |
|  | *OsLPA*2-4R | TCAGGGCTAGAGCACGAAAG |
| Os10g0103800 | *OsITPK*1-1F | CTCTCCTCTCCGCCATTAAAC |
|  | *OsITPK*1-1R | AGACAGCGGCATCAGGTC |
|  | *OsITPK1*-2F | ATGAGGGTGCACGAGGAGGCGT |
|  | *OsITPK1*-2R | TCAGCTCCCGCTTAAACGCCTCTT |
|  | *OsITPK1*-3F | GCACGTCATATATCCTCAAGATCA |
|  | *OsITPK1*-3R | GGGCAATCACATCATTTTGTTG |
| Os03g0230500 | *OsITPK*2-1F | TTCCACTCGTCTCGCCATTAAC |
|  | *OsITPK*2-1R | GAGCCTCGTCACCGGAAC |
|  | *OsITPK*2-2F | ATGCGGCTGCACGGGGAGGTTT |
|  | *OsITPK*2-2R | TCAGCCGCCGCTTAAGCACTTCTT |
|  | *OsITPK*2-3F | TTGTCGTCTCGAATTTCTGTG |
|  | *OsITPK*2-3R | CATTCTAAAAGGATTGCTCATG |
| Os03g0726200 | *OsITPK*3-1F | CTCCCTCTCCCTCTTGCTCTCC |
|  | *OsITPK*3-1R | CTTCGACGTCAGCGCGTA |
|  | *OsITPK*3-2F | ATGGTGTCGGGTGGGCGC |
|  | *OsITPK*3-2R | TCACCCCTCGCATGAGCTATAG |
|  | *OsITPK*3-3F | ACAGAAGTGGCCTTCCTTTG |
|  | *OsITPK*3-3R | AGCAGGTAACATCTTAACTTCA |
| Os10g0576100 | *OsITPK*5-1F | AGCCTGCTTCTCAAAACTACTAACT |
|  | *OsITPK*5-1R | TTCTGGTTTGGACAATTCAGGAATT |
|  | *OsITPK*5-2F | TTCCTGAATTGTCCAAACCAGAATT |
|  | *OsITPK*5-2R | CAGAAAAATTCGTGTTGGTTGGTTC |

| **Gene (RAP-ID)** | **Primer Name** | **Sequence (5'-3')** |
| --- | --- | --- |
| Os09g0518700 | *OsITPK*6-1F | GTGATTCAAAGGGGCTCAAA |
|  | *OsITPK*6-1R | AGCCGTACAAACATTGACCA |
|  | *OsITPK*6-2F | CCTTCCCCTGACATAAAAACCTCTA |
|  | *OsITPK*6-2R | CAATAGCCACTGCATATAAAGACCA |
|  | *OsITPK*6-3F | CCTTTTTAGCATGGTCTATTTGGCA |
|  | *OsITPK*6-3R | TAAATTGTTGCAAAGTGCTCAAGGA |
|  | *OsITPK*6-4F | TCGATTCATTTGGACCTTGCTAGTA |
|  | *OsITPK*6-4R | AAGGGCTTCATCTGTATGCTGATAT |
| Os04g0661200 | *OsIPK*1-1F | ACTTTCCCTCCACGTCTCCT |
|  | *OsIPK*1-1R | ACACCAACTGCTGGGTCCTA |
|  | *OsIPK*1-2F | CCAGGGATGCATGTCCTATG |
|  | *OsIPK*1-2R | GAAACGCCAAGACTTTACCG |
|  | *OsIPK*1-3F | ATCTGTTCCATCCGCAAGAG |
|  | *OsIPK*1-3R | ATTATGGCTGCGGTGAAATC |
|  | *OsIPK*1-4F | GATTTCACCGCAGCCATAAT |
|  | *OsIPK*1-4R | GTGCAAATCAATGGGTTGGT |
|  | *OsIPK*1-5F | ACCAACCCATTGATTTGCAC |
|  | *OsIPK*1-5R | GCATTCGGATGACCTCATTT |
| Os02g0596100 | *OsGLE*1-1F | CGATCTGCTCCGCTGTCTAT |
|  | *OsGLE*1-1R | ATCGTCCTCATCCGTGTCAT |
|  | *OsGLE*1-2F | GCTGCAATCGTTTCCCTCTA |
|  | *OsGLE*1-2R | CCAGAAAATTGCATCACCAA |
|  | *OsGLE*1-3F | TCGACTACAGAAATACGCAGAATC |
|  | *OsGLE*1-3R | CTTCATGGCTTGCTCCTTTC |
|  | *OsGLE*1-4F | CGCAAATTGTGGAACGTAGA |
|  | *OsGLE*1-4R | GTTTTGCATGGTTCCTGGAT |
|  | *OsGLE*1-5F | ATGCAAAACCCATGTTCAGC |
|  | *OsGLE*1-5R | TGTAAGGTCATCATGTAGGCAGTT |
|  | *OsGLE*1-6F | GAACGACATGACCATGCAAT |
|  | *OsGLE*1-6R | GCAAAGCCAGGATAGGAGTG |
|  | *OsGLE*1-7F | CAGCCTAGTTTGCAACCACA |
|  | *OsGLE*1-7R | ACCTGCTACAAGCACACACG |
|  | *OsGLE*1-8F | ATGGCTTGCCATGTTCCTTA |
|  | *OsGLE*1-8R | CTTTATGGCCATCCAGTTCC |
| Os02g0523800 | *OsIPK*2-1F | GCTAGCCGCGCAATCTAC |
|  | *OsIPK*2-1R | GAGGCACTTGGCGACGTA |
|  | *OsIPK*2-2F | CTCTTCTACAAGCCCCTCCA |
|  | *OsIPK*2-2R | AGGCAGTTGGCTCACAATTT |
|  | *OsIPK*2-3F | GCAGTAAACTGTTGTTCCCACA |
|  | *OsIPK*2-3R | CAGAAAAGGCAGGTGTTTGG |

| **Gene (RAP-ID)** | **Primer Name** | **Sequence (5'-3')** |
| --- | --- | --- |
| Os03g0142800 | *OsMRP*13-1F | TCGTAACACGAAGCGAGAGA |
|  | *OsMRP*13-1R | AGGCTAACAATCCCAGCATC |
|  | *OsMRP*13-2F | AATTGGAGTTCACCGACGAC |
|  | *OsMRP*13-2R | GGACATATGCTGCTGATCCA |
|  | *OsMRP*13-3F | GAGGGGTATGCGAGTAGCAG |
|  | *OsMRP*13-3R | CAAGGTCCACGACACTTTGA |
|  | *OsMRP*13-4F | GGTCGAATTTTGAACCGAGT |
|  | *OsMRP*13-4R | TTTAGAGGGGAAAAACACCA |
|  | *OsMRP*13-5F | CCCTGGTCACTTTCAATGCT |
|  | *OsMRP*13-5R | GATTCATCGCTGAACTGCTG |
